# A Rare Autoimmune Hemolytic Overlap: Coombs-Positive Pernicious Anemia

**DOI:** 10.7759/cureus.100566

**Published:** 2026-01-01

**Authors:** Guilherme Ferreira Sacramento, Andrea Castanheira

**Affiliations:** 1 Internal Medicine, Unidade Local de Saúde de Lisboa Ocidental, Lisbon, PRT

**Keywords:** autoimmune hemolytic anemia (aiha), coombs positive anemia, mixed anemia, pernicious-anemia, warm autoimmune hemolytic anemia

## Abstract

Pernicious anemia is a type of autoimmune disease caused by the presence of anti-intrinsic factor and antiparietal cell antibodies, leading to vitamin B12 deficiency and ineffective erythropoiesis. Autoimmune hemolytic anemia is a variant of anemia in which the direct Coombs test is positive. It is uncommon to find a mixed anemia that has both a pernicious anemia component and an autoimmune anemia component with a positive direct Coombs test, as this association is rarely described. Two cases in which this association was found are presented. The first case involved a 65-year-old woman presenting with profound anemia, macrocytosis, reticulocytosis, elevated lactate dehydrogenase, low haptoglobin, and a positive direct Coombs test. She had anti-intrinsic factor antibodies and responded to vitamin B12 supplementation and corticosteroids. The second case involved an 83-year-old woman with severe anemia, vitamin B12 deficiency, positive antiparietal and anti-intrinsic factor antibodies, and positive direct Coombs test, who similarly improved with cobalamin and immunosuppressive therapy. These cases highlight the rare overlap of pernicious anemia and autoimmune hemolytic anemia, emphasizing the importance of recognizing concurrent autoimmune hemolysis in patients with severe vitamin B12 deficiency to guide prompt and appropriate therapy.

## Introduction

Pernicious anemia is an autoimmune disorder characterized by the presence of antiparietal cell antibodies and/or anti-intrinsic factor antibodies, leading to impaired vitamin B12 absorption in the terminal ileum [1]. Vitamin B12 deficiency can result in ineffective erythropoiesis, causing intramedullary hemolysis, which manifests as elevated lactate dehydrogenase (LDH), low haptoglobin, and sometimes indirect hyperbilirubinemia, even in the absence of immune-mediated red cell destruction. Although hemolysis is a recognized feature of cobalamin deficiency, a positive direct antiglobulin (Coombs) test - indicative of true autoimmune hemolytic anemia (AIHA) - is uncommon in this context. Extravascular autoimmune hemolysis is primarily mediated by antibodies that bind to red blood cell surface antigens, leading to phagocytosis and destruction of opsonized erythrocytes outside the circulation [2]. The coexistence of pernicious anemia and AIHA has been reported only in a limited number of case reports, highlighting its rarity [3-5].

Recently, we observed two patients with severe pernicious anemia who also presented with a positive direct antiglobulin test, suggesting the simultaneous presence of both conditions. These cases underscore the diagnostic challenge of distinguishing “pseudo-hemolysis” due to B12 deficiency from true autoimmune hemolysis. We report these cases to emphasize clinical clues that may indicate concurrent AIHA in patients with cobalamin deficiency and to highlight the implications for management, which may require both vitamin B12 replacement and immunosuppressive therapy.

## Case presentation

Case 1

A 65-year-old Black woman from Brazil, with a history of hypertension and type 2 diabetes mellitus, presented to the emergency department with asthenia, persistent lower-limb pain, jaundice, and dyspnea. She denied fever, constitutional symptoms, lymphadenopathy, or overt bleeding. She reported a previous hospitalization 15 years earlier in Brazil for anemia and hepatitis, without further details, and had no known autoimmune diseases. Her medications included B-complex vitamins, antihypertensives, and oral hypoglycemics.

On examination, she was hemodynamically stable but markedly pale and icteric. Laboratory evaluation revealed severe macrocytic anemia (hemoglobin 3.5 g/dL, MCV 123.6 fL) with reticulocytosis (6%), suggesting an adequate marrow response. Hemolysis markers were prominent: markedly elevated LDH (8895 U/L), low haptoglobin (<10 mg/dL), and total bilirubin 2.9 mg/dL (0.77 mg/dL conjugated). Iron studies showed elevated serum iron (244 µg/dL), likely reflecting hemolysis-related iron release due to erythrocyte destruction and ineffective erythropoiesis, and markedly elevated ferritin level (3017 ng/mL), attributable to increased iron storage and its role as an acute-phase reactant in the inflammatory context of the disease.

Vitamin B12 was low (101 pmol/L). Her laboratory evaluation is summarized in Table 1. Peripheral smear demonstrated anisopoikilocytosis, dacryocytes, rare schistocytes, macro-ovalocytes, and hypersegmented neutrophils.

**Table 1:** Laboratory results of the two patients with severe megaloblastic anemia and coexisting warm autoimmune hemolytic anemia. MCV: mean corpuscular volume; MCH: mean corpuscular hemoglobin; RDW: red cell distribution width; LDH: lactate dehydrogenase; N/A: Not available

Test (unit)	Case 1; Observed Value	Case 2; Observed Value	Reference Range	Observations / Clinical Relevance
Hemoglobin (g/dL)	3.5	3.9	12–16	Severe anemia
MCV, fL	123.6	105.7	80–100	Macrocytosis, typical of B12 deficiency, indicative of megaloblastic anemia
MCH, pg	41.2	37.2	27–34	Elevated, supports megaloblastic changes
RDW, %	29.1	44.3	11–15	Compatible with significant anisocytosis, reflecting hemolysis and ineffective erythropoiesis
Leukocytes (×10⁹/L)	11.7	1.3	4–11	Case 2 shows leukopenia, seen in B12 deficiency
Platelets (×10⁹/L)	88	105	150–400	Mild thrombocytopenia, often seen in B12 deficiency
Reticulocyte count (%)	6	N/A	0.5–1.5	Case 1: reticulocytosis indicates compensatory marrow response, supporting concomitant hemolysis
LDH, U/L	8895	3753	140–280	Markedly elevated due to intramedullary hemolysis and autoimmune hemolysis
Haptoglobin (mg/dL)	<10	12	50–220	Low, indicative of hemolysis
Total bilirubin (mg/dL)	2.9	2.1	<0.9	Mild indirect hyperbilirubinemia due to hemolysis
Iron (µg/dL)	244	192	50–170	Elevated, reflecting erythrocyte destruction and ineffective erythropoiesis, and iron overload
Ferritin (ng/mL)	3017	957	10–120	Elevated due to increased iron stores from ineffective erythropoiesis / acute phase reactant
Vitamin B12 (pmol/L)	101	<74	141–489	Deficient in both cases
Folate (nmol/L)	20	20.2	10–42	Within normal range
Antiparietal cell antibodies	Negative	Positive	-	Supports autoimmune etiology of B12 deficiency
Anti-intrinsic factor antibodies	Positive	Positive	-	Confirms diagnosis of pernicious anemia
Direct Coombs test	Positive (IgG)	Positive (IgG)	-	Confirms presence of warm autoimmune hemolytic anemia

Given the degree of hemolysis and reticulocytosis disproportionate to isolated vitamin B12 deficiency, a direct Coombs test was performed and was positive for IgG, confirming warm AIHA. Anti-intrinsic factor antibodies were detected, establishing pernicious anemia as the cause of vitamin B12 deficiency. Viral serologies were consistent with past hepatitis B and Epstein-Barr virus infection, while HIV and HCV testing were negative. Complement analysis revealed isolated low C3 levels (76.3 mg/dL). Serum protein electrophoresis showed no monoclonal component, and antiplatelet antibody testing was negative, helping to exclude hematologic malignancy, active viral infection, or secondary autoimmune processes. The patient was not taking any concomitant medications known to induce AIHA.

The patient received three units of packed red blood cells and was started on prednisolone 60 mg/day (1 mg/kg) along with subcutaneous vitamin B12 injections daily for one week, followed by weekly dosing. Within one week, hemoglobin rose to 9.5 g/dL, LDH decreased to 1896 U/L, and reticulocyte count remained elevated at 5.5%, consistent with concurrent AIHA. At the three-month follow-up, hemoglobin was stable at 12.7 g/dL, LDH was almost normalized (275 U/L), haptoglobin was normal (149 mg/dL), and corticosteroids were tapered without relapse.

Case 2

An 83-year-old Black woman with hypertension and a delusional disorder was admitted with severe anemia. Her clinical history was limited. Laboratory evaluation revealed hemoglobin 3.9 g/dL, MCV 105.7 fL, leukocytes 1.3×10⁹/L, platelets 105×10⁹/L, LDH 3753 U/L, iron 192 µg/dL, ferritin 957 ng/mL, transferrin saturation 90%, folate 20.2 nmol/L, and vitamin B12 < 74 pmol/L (Table 1). Both antiparietal cell and anti-intrinsic factor antibodies were positive. Endoscopic evaluation was refused.

Her direct Coombs test was also positive for IgG. Autoimmune panel and viral serologies (HIV, HCV, HBV, EBV) were negative. She received two units of packed red blood cells, cyanocobalamin injections, and prednisolone. She was discharged with a stable hemoglobin level of 9.1 g/dL.

After discharge, the patient was scheduled for outpatient follow-up to monitor hematologic response and continue vitamin B12 supplementation and corticosteroid tapering. However, she did not attend the planned follow-up appointments and was subsequently lost to hospital follow-up, precluding further longitudinal assessment of treatment response or disease course.

## Discussion

Pernicious anemia results from autoimmune destruction of gastric parietal cells and/or neutralization of intrinsic factor, leading to reduced cobalamin absorption and eventual deficiency [1]. The diagnosis is supported by low vitamin B12 levels and the presence of autoantibodies, and is often confirmed by endoscopic evidence of atrophic gastritis [1].

Cobalamin deficiency can present with hemolytic features - elevated LDH, low haptoglobin, and indirect hyperbilirubinemia - due to ineffective erythropoiesis causing intramedullary hemolysis [3].

Hemolytic features in B12 deficiency are likely under-recognized. Case reports and series have documented overt hemolysis in severe cobalamin deficiency, even without direct antiglobulin positivity, suggesting mechanisms such as ineffective erythropoiesis, hyperhomocysteinemia-induced red cell fragility, and impaired red cell deformability [4]. Several recent reports describe patients with pernicious anemia presenting with severe hemolysis, pancytopenia, or both [5-9]. However, the finding of a positive direct Coombs test, indicative of AIHA, is highly unusual in the context of pernicious anemia [3,4].

Warm AIHA is characterized by IgG-mediated erythrocyte destruction and is typically idiopathic or secondary to hematologic malignancies, autoimmune disorders, or medications such as penicillins [2,10]. Vitamin B12 deficiency is not recognized as an underlying cause.

The coexistence of pernicious anemia and warm AIHA, although rare, has been reported. Recent cases have documented patients with severe vitamin B12 deficiency and confirmed IgG-mediated hemolysis who required both cobalamin replacement and immunosuppressive therapy to achieve hematologic recovery [3,4]. In some instances, the direct antiglobulin test initially was negative but became positive following B12 supplementation, highlighting the dynamic interplay between cobalamin deficiency and autoimmune hemolysis [4].

In the cases presented, the combination of severe macrocytosis, hypersegmented neutrophils, anti-intrinsic factor antibodies, and low B12 indicated pernicious anemia. However, the disproportionate hemolysis, high reticulocytosis, and positive Coombs test confirmed concurrent AIHA. Alternative causes of hemolysis, including infection, drug-induced hemolysis, and malignancy, were excluded.

The simultaneous presence of severe vitamin B12 deficiency and warm AIHA in the two unrelated patients from the cases highlights a rare but potentially relevant association. Whether this represents a true pathophysiologic link or a coincidental overlap of autoimmune processes remains unclear. Nonetheless, distinguishing the two entities is clinically important because management differs: pernicious anemia requires B12 replacement, whereas AIHA requires immunosuppression [11]. In both cases, corticosteroid therapy was initiated due to confirmed warm AIHA in addition to vitamin B12 deficiency.

We believe it is important to present these cases, since this association has been discussed very infrequently in the literature. Its description may be important in helping to understand a relationship that is still poorly understood.

## Conclusions

These two cases illustrate the rare coexistence of pernicious anemia and IgG-mediated autoimmune hemolytic anemia confirmed by positive direct Coombs tests, severe vitamin B12 deficiency with autoantibody positivity, and biochemical markers of hemolysis (elevated LDH, low haptoglobin, and reticulocytosis). While B12 deficiency alone can cause intramedullary hemolysis, the disproportionate hemolysis and positive Coombs tests, along with the favorable response to combined cobalamin replacement and corticosteroid therapy, support the presence of a concurrent autoimmune mechanism.

These findings underscore the clinical importance of considering AIHA in patients with pernicious anemia who present with profound anemia or unexpectedly severe hemolysis, as timely recognition guides both B12 supplementation and immunosuppressive therapy.

Importantly, this report is based on case-level evidence and is, therefore, primarily hypothesis-generating, highlighting an association that is rare or possibly underrecognized. Further studies are needed to clarify whether a true pathophysiological link exists between pernicious anemia and AIHA, or whether these represent coincidental but distinct autoimmune processes.
